# Synthesis, crystal structure and *in vitro* anti-proliferative activity of 2-[(4-acetyl­phen­yl)carbamo­yl]phenyl acetate

**DOI:** 10.1107/S2056989023008526

**Published:** 2023-10-05

**Authors:** Reham A. Mohamed-Ezzat, Benson M. Kariuki, Aladdin M. Srour

**Affiliations:** a Chemistry of Natural & Microbial Products Department, National Research Centre, Cairo, Egypt; b School of Chemistry, Cardiff University, Main Building, Park Place, Cardiff CF10, 3AT, United Kingdom; c Department of Therapeutic Chemistry, National Research Centre, Dokki, Cairo, 12622, Egypt; Universidad de Los Andes Mérida, Venezuela

**Keywords:** crystal structure, aspirin derivative, NCI 60 cell line, anti-proliferative activity

## Abstract

2-[(4-Acetyl­phen­yl)carbamo­yl]phenyl acetate, C_17_H_15_NO_4_, has been synthesized and structurally characterized. In the structure, N—H⋯O hydrogen-bonding inter­actions form chains of mol­ecules aligned along the [101] direction. The chains are linked by π–π and C—H⋯π inter­actions, forming a three dimensional network. The compound has been screened for *in vitro* anti-proliferative activity revealing considerable activity.

## Abstract 2

2-[(4-Acetyl­phen­yl)carbamo­yl]phenyl acetate, a derivative of aspirin, has been structurally characterized revealing a structure based on inter­molecular N—H⋯O hydrogen bonds and π–π inter­actions.

## 1. Chemical context

Acetyl­salicylic acid (ASA), or aspirin, is a non-steroidal anti-inflammatory drug (NSAID) utilized extensively as an analgesic and anti­pyretic agent. It has been shown to induce apoptotic cell death in several cancer cell lines **(**Brune & Patrignani, 2015 ▸; Ranger *et al.*, 2020 ▸; Abd-El-Aziz *et al.*, 2021 ▸). Aspirin is one of the most prescribed drugs for pain relief as well as for cardiovascular prophylaxis. Decades of investigations have provided substantial evidence indicating potential in the prevention of cancer, particularly colorectal cancer (Drew *et al.*, 2016 ▸). Comprehensive clinical benefits of aspirin-based chemoprevention strategies have lately been acknowledged. However, due to the identified risks of long-term aspirin usage, larger scale adoption of an aspirin chemoprevention strategy is likely to involve enhanced identification of individuals for whom the protective benefits compensate the side effects (Drew *et al.*, 2016 ▸). Aspirin is recognized as a means for prevention of ischemic heart attack and stroke (Pinto *et al.*, 2013 ▸). Although several effects of aspirin are related to its ability to inhibit cyclo­oxygenase (COX), a key enzyme in prostaglandin biosynthesis, COX-independent effects have also been reported (Alfonso *et al.*, 2014 ▸). Aspirin has emerged as a promising inter­vention in cancer treatment in the past decade (Tran *et al.*, 2021 ▸; Lichtenberger *et al.*, 2019 ▸), and has a protective effect against several types of cancer (Garcia-Albeniz *et al.*, 2011 ▸; Usman *et al.*, 2015 ▸). It induces cell death in various cancer cell lines, such as myeloid leukaemia and HeLa cells, chronic lymphocytic leukaemia cells, colon cancer cells (Bellosillo *et al.*, 1998 ▸), gastric cancer (Gu *et al.*, 2005 ▸), colorectal cancer (Stark *et al.*, 2007 ▸) and cholangiocarcinoma (Shen & Shen, 2021 ▸).

Motivated by the properties enumerated above and in continuation of our inter­est in the synthesis of aspirin-based scaffolds, 2-[(4-acetyl­phen­yl)carbamo­yl]phenyl acetate was synthesized and characterized. It was anti­cipated that the compound would present biological activity and it was tested against an NCI 60 cell-line panel.

**Figure 1:**
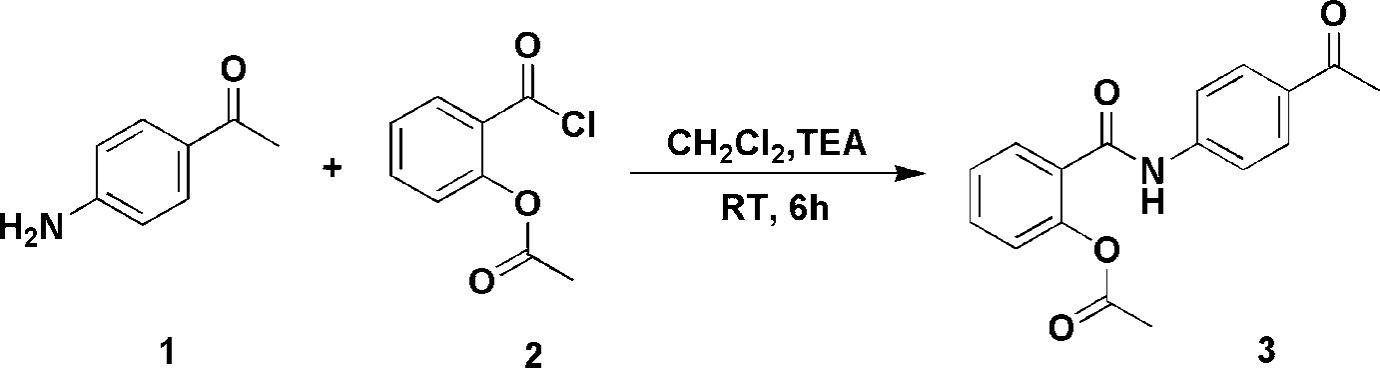
Synthetic route towards compound **3**.

Facile synthesis of the target 2-[(4-acetyl­phen­yl)carbamo­yl]phenyl acetate (**3**) was carried out through the reaction of 4*’*-amino aceto­phenone (**1**) and 2-(chloro­carbon­yl)phenyl acetate (**2**) in the presence of a qu­anti­tative amount of triethyl amine (Fig. 1 ▸). The chemical identity of the product was confirmed by various spectroscopic techniques consistent with literature reports (Gao *et al.*, 2014 ▸; Eissa *et al.*, 2017 ▸). 
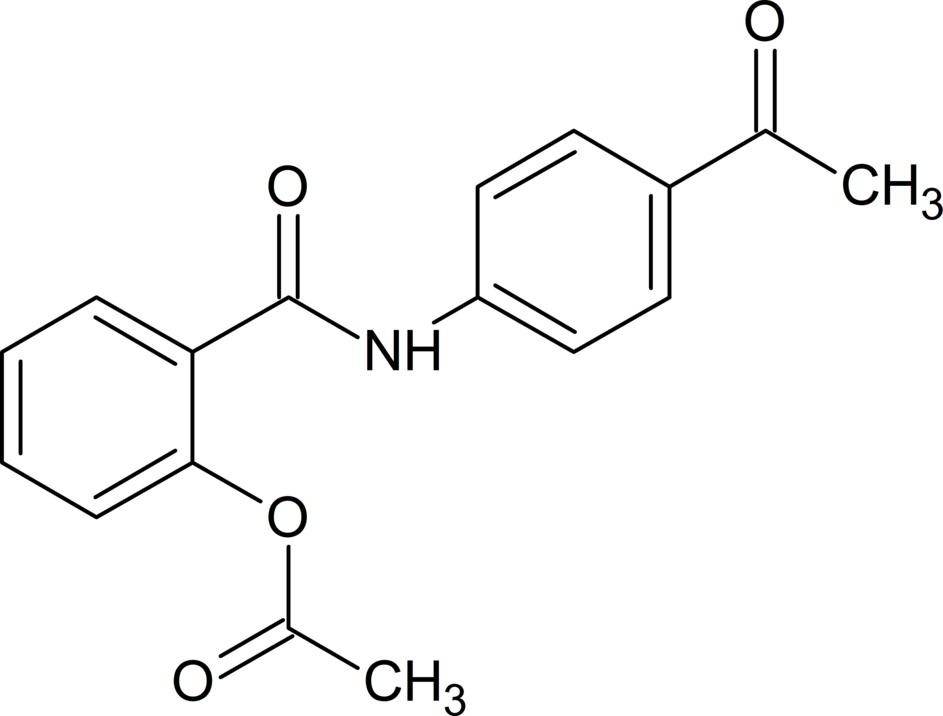


## 2. Structural commentary

**Figure 2:**
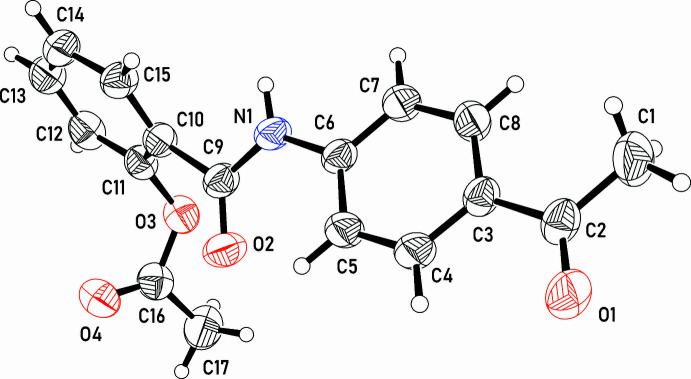
The asymmetric unit of (**3**) showing displacement ellipsoids at the 50% probability level.

The asymmetric unit is shown in Fig. 2 ▸. The phenyl­ethanone fragment of the mol­ecule is essentially planar with a twist angle between the phenyl ring (C3–C8) and the acetaldehyde group (C1,C2,O1) of 4.7 (2)°. In the phenyl­acetate group of the mol­ecule, the acetate group (C16,C17,O3,O4) is almost perpendicular to the phenyl ring (C10–C15) with a twist angle of 82.39 (6)°. This relationship between the acetate group and the ring is similar to that found in aspirin (Tyler *et al.*, 2020 ▸). The formamide group (C9,N1,O2) is twisted by 25.14 (14)° from one phenyl ring (C3–C8) and by 45.53 (8)° from the second (C10–C15). There is an intra­molecular C5—H5⋯O2 hydrogen bond (Table 1 ▸).

The twist angles between the various groups in the mol­ecule are similar to those of the *N*-(4-acetyl­phen­yl)benzamide moiety in *N*-(4-acetyl­phen­yl)-2-[(1,3-dioxo-1,3-di­hydro-2*H*-isoindol-2-yl)meth­yl]benzamide (Mourad *et al.*, 2020 ▸).

## 3. Supra­molecular features

**Figure 3:**
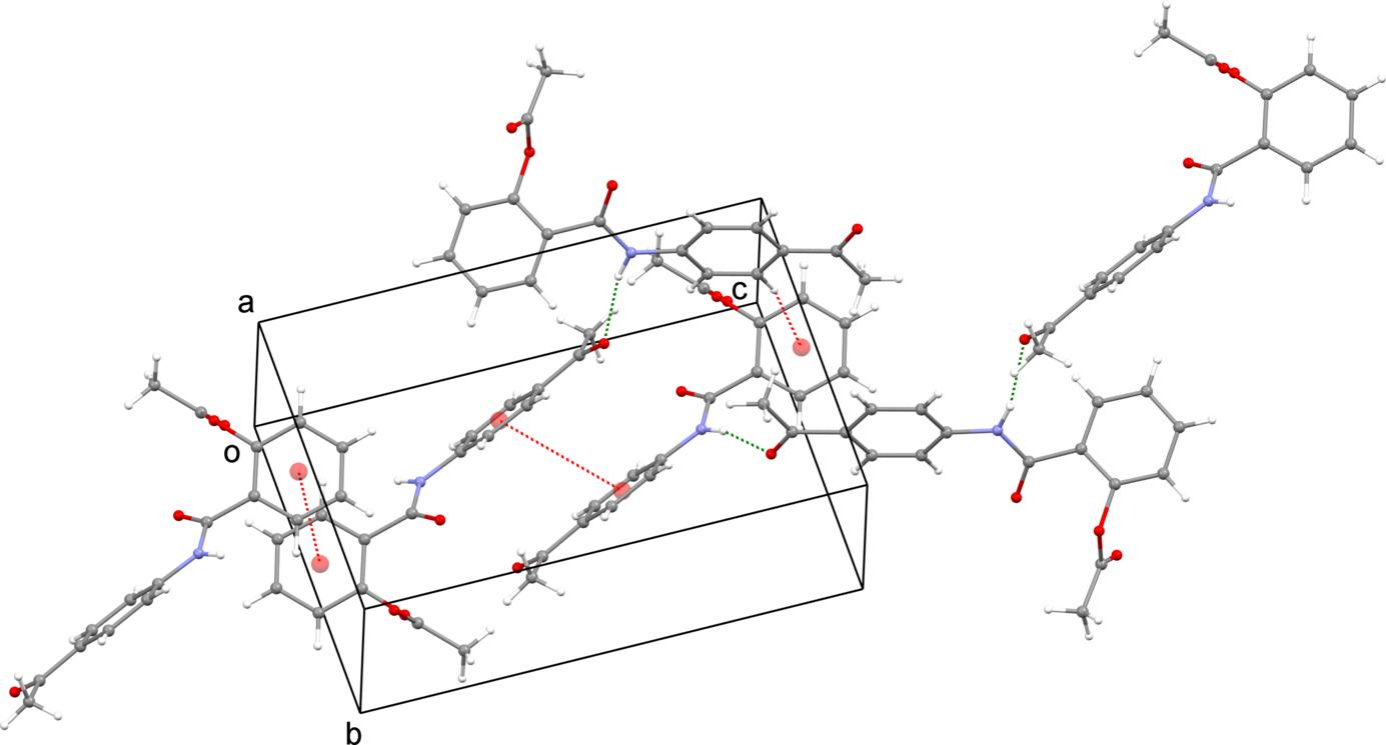
A segment of the crystal structure of compound **3** showing inter­molecular contacts (N—H⋯O in green, π–π and C—H⋯π in red).

In the crystal, N—H⋯O hydrogen-bonding inter­actions occur between neighbouring mol­ecules related by − 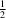 + *x*, 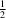 − *y*, − 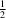 + *z*, resulting in chains parallel to the [101] direction (Fig. 3 ▸, Table 1 ▸). A C12—H12⋯O4^i^ hydrogen bond is also observed. Contacts of the type π–π are also observed between symmetry-related phenyl rings from neighbouring mol­ecules with centroid-to-centroid distances of 4.0823 (9) Å (C3–C8 rings, symmetry operation 1 − *x*, 1 − *y*, 1 − *z*) and 3.9417 (10) Å (C10–C15 rings, symmetry operation 1 − *x*, 1 − *y*, −*z*). Additionally, a C—H⋯π inter­action occurs between the edge of the C3–C8 ring and the face of the C10–C15 ring (Table 1 ▸).

## 4. Database survey

A search of the Cambridge Structural Database (Version 5.43, update of November, 2022; Groom *et al.*, 2016 ▸) for structures containing the *N*-(4-acetyl­phen­yl)benzamide moiety pro­duced one hit for *N*-(4-acetyl­phen­yl)-2-[(1,3-dioxo-1,3-di­hydro-2*H*-isoindol-2-yl)meth­yl]benzamide (LACYIB; Mourad *et al.*, 2020 ▸).

## 5. Synthesis and crystallization

Melting points were determined on a Stuart SMP30 melting-point apparatus. IR spectra (KBr) were recorded on a JASCO 6100 spectrophotometer. NMR spectra were recorded on a JEOL AS 500 (DMSO-*d*_6_, ^1^H: 500 MHz, ^13^C: 125 MHz) spectrometer, JEOL USA, Inc. Mass spectra were recorded on a Shimadzu GCMS-QP 1000 EX (EI, 70 eV) spectrometer, Shimadzu Corporation, Kyoto, Japan. Elemental micro analyses were performed using a Vario Elemental analyzer, Elementar Analysensysteme GmbH, Langenselbold, Germany. Figs. S1–S4 of the Supplementary material show the spectroscopic data. The starting compound 2-(chloro­carbon­yl)phenyl acetate (**1**) was prepared according to previously reported procedures (Sharma *et al.*, 2011 ▸; Ngaini *et al.*, 2012 ▸).

*Synthesis of 2-[(4-acetyl­phen­yl)carbamo­yl]phenyl acetate (**3**)*

To a stirred solution of 4′-amino­aceto­phenone (**1**) (1.35 g, 10 mmol) and triethyl amine (1.48 ml, 11 mmol) in 25 ml of di­chloro­methane, 2-(chloro­carbon­yl)phenyl acetate (**2**) (1.98 g, 10 mmol) was added portion-wise over a period of 30 min, and the mixture was stirred at room temperature for 6 h (Fig. 1 ▸). The mixture was filtered, the solvent evaporated under reduced pressure, and then the solid obtained was washed with water, dried and recrystallized from benzene/pet. ether 60–80.

Buff-colored crystals; yield (2.65 g) 89%; mp 424–426 K; IR (*ν*_max_/cm^−1^): 3297 (NH), 1659, 1679, 1760 (C=O); ^1^H NMR (DMSO-*d*_6_) δ (ppm): 2.18 (*s*, 3H, CH_3_), 2.52 (*s*, 3H, COCH_3_), 7.24, 7.26 (*dd*, 1H, *J* = 1.20, 1.10 Hz, CH), 7.39 (*t*, 1H, *J* = 8.13 Hz, CH), 7.57 (*t*, 1H, *J* = 8.65 Hz, CH), 7.70, 7.71 (*dd*, 1H, *J* = 1.65, 1.65 Hz, CH), 7.85 (*d*, 2H, *J* = 8.8 Hz, CH), 7.94 (*d*, 2H, *J* = 8.8 Hz, CH), 10.71 (*s*, 1H, NH); ^13^C NMR (DMSO-*d*_6_) δ (ppm) 20.84, 26.60, 119.28, 123.48, 126.08, 129.37, 129.45, 129.53, 129.57, 132.03, 132.32, 143.64, 148.30, 164.80, 169.04, 196.74; MS: m/z (%) 297.88 (M^+^, 9.97); Analysis calculated for C_17_H_15_NO_4_ (297.31): C, 68.68; H, 5.09; N, 4.71. Found: C, 68.66; H, 5.10; N, 4.70.

## 6. Refinement

Crystal data, data collection and structure refinement details are summarized in Table 2 ▸. H atoms were positioned geometrically and refined as riding with *U*_iso_(H) = 1.2 or 1.5*U*_eq_(C,N).

## 7. *In vitro* anti-proliferative activity against NCI 60 cell-lines panel

The title compound was selected by the National Cancer Institute (NCI), NIH, USA under the Developmental Therapeutic Program (DTP) for the estimation of *in vitro* anti­proliferative activity against the NCI 60 cell-line panel. This screen utilizes human tumour cell lines, representing melanoma, leukemia, colon, lung, ovary, brain, prostate, kidney and breast cancers.

The NCI screening service ranks compounds with a promising drug-like mode of action on the basis of computer-aided design. The capability of the submitted compounds to convey diversity to the NCI small mol­ecule compound collection is critical to selecting them for screening.

**Figure 4:**
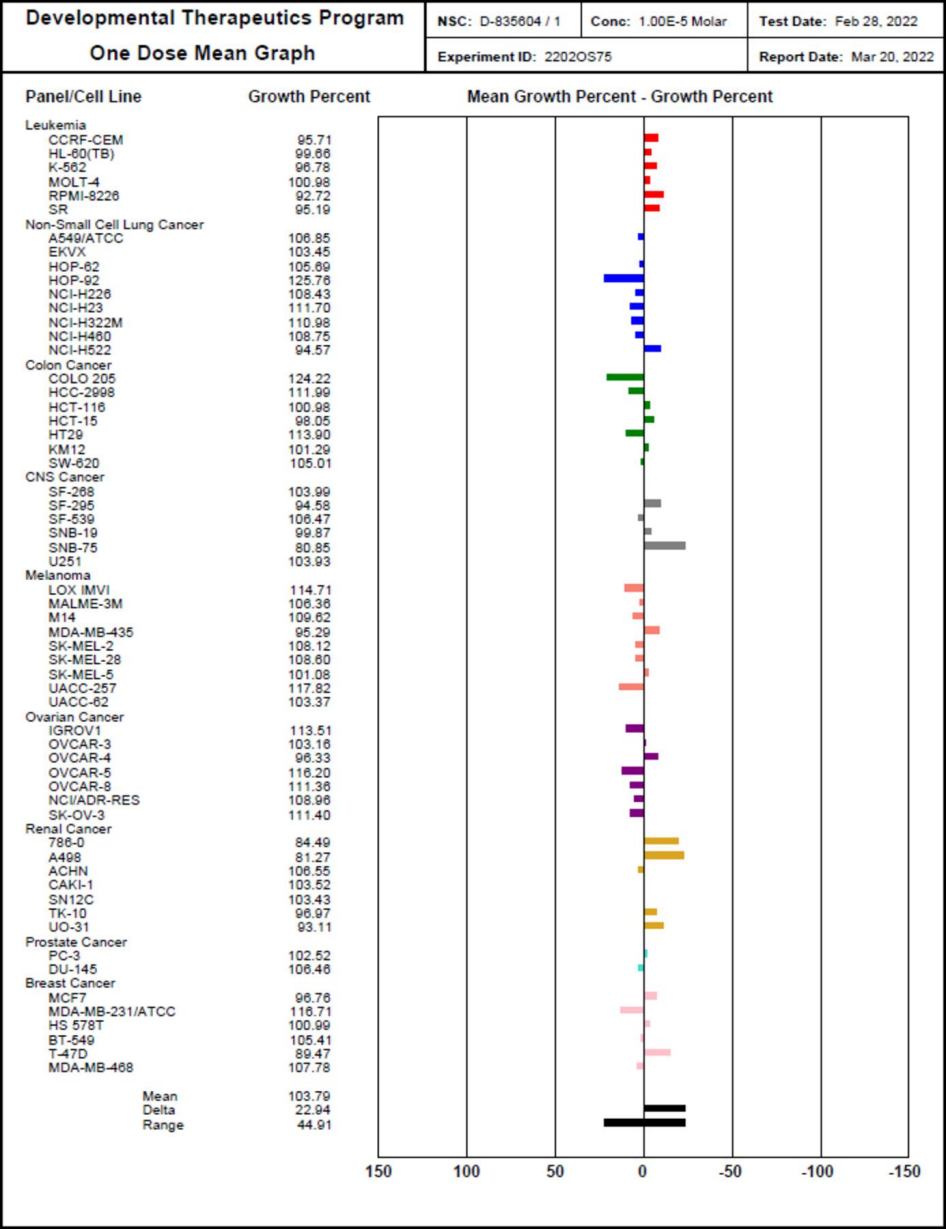
*In vitro* anti-proliferative activity data of compound **3** at 10^−5^ *M*.

The title compound was assigned NCI code NSC D-832401 representing the chemotype of this work. It was screened at an initial 10 *μ*M one-dose % inhibition assay on the full NCI 60 cell-line panel. The results are shown in Fig. 4 ▸. The results are represented as cell growth % for the compound in each of the panels. The lowest cell-growth promotion was observed on leukemia RPMI-8226 cell line (GP = 92.72%), non-small-cell lung cancer NCI-H522 (GP = 94.57%), colon cancer HCT-15 (GP = 98.05%), CNS cancer SNB-75 (GP = 80.85%), melanoma MDA-MB-43 (GP = 95.29%), ovarian cancer OVCAR-4 (GP = 96.33%), renal cancer A498 (GP = 81.27%) and breast cancer T-47D (GP = 89.47%).

Thus, in general, the compound displays considerable *in vitro* anti-proliferative activity at 10 *μ*M against most of the tested cancer cell lines. This supports possible future experiments on this compound including the determination of IC^50^ (for the most promising cell line) and cytotoxicity in normal cells.

## Supplementary Material

Crystal structure: contains datablock(s) I. DOI: 10.1107/S2056989023008526/dj2060sup1.cif

Structure factors: contains datablock(s) I. DOI: 10.1107/S2056989023008526/dj2060Isup2.hkl

Supporting information file. DOI: 10.1107/S2056989023008526/dj2060Isup3.cml

Supporting information file. DOI: 10.1107/S2056989023008526/dj2060sup4.docx

CCDC reference: 2087303

Additional supporting information:  crystallographic information; 3D view; checkCIF report

## Figures and Tables

**Table 1:** Hydrogen-bond geometry (Å, °) *Cg*1 is the centroid of the C10–C15 ring.

*D*—H⋯*A*	*D*—H	H⋯*A*	*D*⋯*A*	*D*—H⋯*A*
C5—H5⋯O2	0.93	2.35	2.8792 (19)	116
C12—H12⋯O4^i^	0.93	2.59	3.396 (2)	145
N1—H1⋯O1^ii^	0.86	2.08	2.9181 (16)	164
C8—H8⋯*Cg*1^iii^	0.93	3.20	4.0960 (15)	164

Symmetry codes: (i) 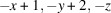 ; (ii) 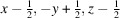 ; (iii) 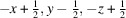 .

**Table 2:** Experimental details

Crystal data	
Chemical formula	C_17_H_15_NO_4_
*M*_r_	297.30
Crystal system, space group	Monoclinic, *P*2_1_/*n*
Temperature (K)	296
*a*, *b*, *c* (Å)	11.6286 (5), 8.6913 (4), 15.8180 (7)
β (°)	110.380 (5)
*V* (Å^3^)	1498.62 (12)
*Z*	4
Radiation type	Cu *K*α
μ (mm^−1^)	0.78
Crystal size (mm)	0.35 × 0.14 × 0.13

Data collection	
Diffractometer	Agilent SuperNova, Dual, Cu at home/near, Atlas
Absorption correction	Gaussian (*CrysAlis PRO*; Rigaku OD, 2019 ▸)
*T*_min_, *T*_max_	0.390, 1.000
No. of measured, independent and observed [*I* > 2σ(*I*)] reflections	17065, 3141, 2612
*R*_int_	0.027
(sin θ/λ)_max_ (Å^−1^)	0.632

Refinement	
*R*[*F* ^2^ > 2σ(*F* ^2^)], *wR*(*F* ^2^), *S*	0.043, 0.134, 1.03
No. of reflections	3141
No. of parameters	201
H-atom treatment	H-atom parameters constrained
Δρ_max_, Δρ_min_ (e Å^−3^)	0.17, −0.16

Computer programs: *CrysAlis PRO* (Rigaku OD, 2019 ▸), *SHELXS* (Sheldrick, 2008 ▸), *SHELXL2019/3* (Sheldrick, 2015 ▸), *ORTEP-3 for Windows* (Farrugia, 2012 ▸) and *Mercury* (Macrae *et al.*, 2020 ▸).

## supplementary crystallographic information

### Crystal data

**Table:**

C_17_H_15_NO_4_	*F*(000) = 624
*M**_r_* = 297.30	*D*_x_ = 1.318 Mg m^−^^3^
Monoclinic, *P*2_1_/*n*	Cu *K*α radiation, λ = 1.54184 Å
*a* = 11.6286 (5) Å	Cell parameters from 5304 reflections
*b* = 8.6913 (4) Å	θ = 4.1–76.2°
*c* = 15.8180 (7) Å	µ = 0.78 mm^−^^1^
β = 110.380 (5)°	*T* = 296 K
*V* = 1498.62 (12) Å^3^	Needle, yellow
*Z* = 4	0.35 × 0.14 × 0.13 mm

### Data collection

**Table:**

Agilent SuperNova, Dual, Cu at home/near, Atlas diffractometer	2612 reflections with *I* > 2σ(*I*)
ω scans	*R*_int_ = 0.027
Absorption correction: gaussian (CrysAlisPro; Rigaku OD, 2019)	θ_max_ = 76.9°, θ_min_ = 4.1°
*T*_min_ = 0.390, *T*_max_ = 1.000	*h* = −14→14
17065 measured reflections	*k* = −10→10
3141 independent reflections	*l* = −19→19

### Refinement

**Table:**

Refinement on *F*^2^	Primary atom site location: structure-invariant direct methods
Least-squares matrix: full	Hydrogen site location: inferred from neighbouring sites
*R*[*F*^2^ > 2σ(*F*^2^)] = 0.043	H-atom parameters constrained
*wR*(*F*^2^) = 0.134	*w* = 1/[σ^2^(*F*_o_^2^) + (0.0761*P*)^2^ + 0.1892*P*] where *P* = (*F*_o_^2^ + 2*F*_c_^2^)/3
*S* = 1.03	(Δ/σ)_max_ = 0.002
3141 reflections	Δρ_max_ = 0.17 e Å^−^^3^
201 parameters	Δρ_min_ = −0.16 e Å^−^^3^
0 restraints	

### Special details

**Table:**

Geometry. All esds (except the esd in the dihedral angle between two l.s. planes) are estimated using the full covariance matrix. The cell esds are taken into account individually in the estimation of esds in distances, angles and torsion angles; correlations between esds in cell parameters are only used when they are defined by crystal symmetry. An approximate (isotropic) treatment of cell esds is used for estimating esds involving l.s. planes.

### Fractional atomic coordinates and isotropic or equivalent isotropic displacement parameters (Å^2^)

**Table:**

	*x*	*y*	*z*	*U*_iso_*/*U*_eq_	
C1	0.5388 (2)	0.1466 (3)	0.63141 (13)	0.0868 (6)	
H1A	0.587598	0.105244	0.689002	0.130*	
H1B	0.498487	0.064212	0.591739	0.130*	
H1C	0.478372	0.215398	0.638833	0.130*	
C2	0.61923 (14)	0.23211 (19)	0.59205 (9)	0.0576 (4)	
C3	0.56774 (12)	0.29253 (17)	0.49855 (9)	0.0505 (3)	
C4	0.64622 (13)	0.36063 (19)	0.46058 (10)	0.0587 (4)	
H4	0.728942	0.370691	0.495050	0.070*	
C5	0.60455 (13)	0.41363 (19)	0.37314 (10)	0.0589 (4)	
H5	0.658915	0.457351	0.348638	0.071*	
C6	0.48068 (12)	0.40141 (16)	0.32165 (8)	0.0490 (3)	
C7	0.40109 (13)	0.3340 (2)	0.35836 (10)	0.0624 (4)	
H7	0.318208	0.325327	0.324040	0.075*	
C8	0.44443 (13)	0.2795 (2)	0.44606 (10)	0.0619 (4)	
H8	0.390386	0.233633	0.470096	0.074*	
C9	0.48064 (13)	0.56038 (17)	0.19145 (9)	0.0539 (3)	
C10	0.41025 (12)	0.58347 (17)	0.09278 (9)	0.0512 (3)	
C11	0.38681 (13)	0.73080 (17)	0.05690 (10)	0.0532 (3)	
C12	0.32771 (14)	0.7548 (2)	−0.03390 (11)	0.0629 (4)	
H12	0.311768	0.854402	−0.056485	0.075*	
C13	0.29218 (15)	0.6303 (2)	−0.09134 (10)	0.0668 (4)	
H13	0.252995	0.646157	−0.152872	0.080*	
C14	0.31454 (15)	0.4827 (2)	−0.05786 (10)	0.0634 (4)	
H14	0.291076	0.398867	−0.096645	0.076*	
C15	0.37213 (14)	0.45989 (19)	0.03382 (10)	0.0574 (4)	
H15	0.385567	0.360130	0.056397	0.069*	
C16	0.52387 (15)	0.93013 (18)	0.12475 (11)	0.0606 (4)	
C17	0.5436 (2)	1.0612 (2)	0.18968 (14)	0.0858 (6)	
H17A	0.559486	1.021628	0.249346	0.129*	
H17B	0.471610	1.124674	0.172543	0.129*	
H17C	0.612495	1.121222	0.188800	0.129*	
N1	0.43273 (11)	0.45087 (15)	0.23117 (7)	0.0547 (3)	
H1	0.366161	0.407257	0.197490	0.066*	
O1	0.72755 (11)	0.25066 (17)	0.63648 (8)	0.0776 (4)	
O2	0.57285 (12)	0.63430 (15)	0.23103 (8)	0.0745 (4)	
O3	0.41633 (10)	0.85821 (13)	0.11491 (8)	0.0629 (3)	
O4	0.58982 (12)	0.89231 (15)	0.08529 (9)	0.0739 (3)	

### Atomic displacement parameters (Å^2^)

**Table:**

	*U*^11^	*U*^22^	*U*^33^	*U*^12^	*U*^13^	*U*^23^
C1	0.0814 (12)	0.1205 (18)	0.0571 (9)	0.0023 (11)	0.0223 (9)	0.0268 (10)
C2	0.0559 (8)	0.0687 (9)	0.0445 (7)	0.0146 (6)	0.0127 (6)	0.0053 (6)
C3	0.0473 (7)	0.0592 (8)	0.0412 (6)	0.0072 (5)	0.0108 (5)	0.0026 (5)
C4	0.0424 (7)	0.0737 (10)	0.0497 (7)	−0.0019 (6)	0.0030 (5)	0.0093 (6)
C5	0.0461 (7)	0.0745 (10)	0.0496 (7)	−0.0079 (6)	0.0087 (6)	0.0102 (6)
C6	0.0478 (7)	0.0533 (7)	0.0391 (6)	−0.0021 (5)	0.0067 (5)	0.0010 (5)
C7	0.0422 (7)	0.0892 (11)	0.0470 (7)	−0.0061 (7)	0.0045 (5)	0.0094 (7)
C8	0.0470 (7)	0.0872 (11)	0.0497 (7)	−0.0017 (7)	0.0146 (6)	0.0110 (7)
C9	0.0555 (7)	0.0560 (8)	0.0444 (7)	−0.0038 (6)	0.0101 (6)	0.0016 (5)
C10	0.0477 (7)	0.0579 (8)	0.0452 (7)	−0.0002 (5)	0.0127 (5)	0.0061 (6)
C11	0.0479 (7)	0.0588 (8)	0.0545 (7)	0.0020 (6)	0.0198 (6)	0.0055 (6)
C12	0.0563 (8)	0.0691 (10)	0.0619 (9)	0.0089 (7)	0.0190 (7)	0.0216 (7)
C13	0.0589 (8)	0.0881 (12)	0.0467 (7)	−0.0016 (8)	0.0102 (6)	0.0154 (7)
C14	0.0633 (9)	0.0749 (10)	0.0465 (7)	−0.0083 (7)	0.0122 (6)	−0.0003 (7)
C15	0.0595 (8)	0.0576 (8)	0.0495 (7)	−0.0028 (6)	0.0118 (6)	0.0045 (6)
C16	0.0676 (9)	0.0522 (8)	0.0618 (8)	0.0008 (6)	0.0223 (7)	0.0114 (6)
C17	0.1228 (17)	0.0633 (10)	0.0751 (12)	−0.0140 (11)	0.0391 (12)	−0.0023 (9)
N1	0.0501 (6)	0.0636 (7)	0.0401 (6)	−0.0099 (5)	0.0029 (4)	0.0047 (5)
O1	0.0566 (7)	0.1113 (11)	0.0531 (6)	0.0140 (6)	0.0042 (5)	0.0188 (6)
O2	0.0769 (8)	0.0774 (8)	0.0537 (6)	−0.0279 (6)	0.0031 (5)	0.0077 (5)
O3	0.0680 (7)	0.0566 (6)	0.0695 (7)	0.0020 (5)	0.0307 (5)	0.0010 (5)
O4	0.0680 (7)	0.0697 (8)	0.0905 (9)	−0.0052 (5)	0.0356 (7)	0.0014 (6)

### Geometric parameters (Å, º)

**Table:**

C1—C2	1.490 (3)	C9—C10	1.5022 (18)
C1—H1A	0.9600	C10—C11	1.389 (2)
C1—H1B	0.9600	C10—C15	1.390 (2)
C1—H1C	0.9600	C11—C12	1.375 (2)
C2—O1	1.221 (2)	C11—O3	1.4024 (18)
C2—C3	1.4848 (18)	C12—C13	1.380 (3)
C3—C4	1.388 (2)	C12—H12	0.9300
C3—C8	1.3890 (19)	C13—C14	1.378 (2)
C4—C5	1.3759 (19)	C13—H13	0.9300
C4—H4	0.9300	C14—C15	1.384 (2)
C5—C6	1.3902 (19)	C14—H14	0.9300
C5—H5	0.9300	C15—H15	0.9300
C6—C7	1.382 (2)	C16—O4	1.191 (2)
C6—N1	1.4101 (16)	C16—O3	1.358 (2)
C7—C8	1.384 (2)	C16—C17	1.497 (3)
C7—H7	0.9300	C17—H17A	0.9600
C8—H8	0.9300	C17—H17B	0.9600
C9—O2	1.2194 (18)	C17—H17C	0.9600
C9—N1	1.3619 (19)	N1—H1	0.8600
			
C2—C1—H1A	109.5	C11—C10—C9	120.41 (13)
C2—C1—H1B	109.5	C15—C10—C9	121.66 (13)
H1A—C1—H1B	109.5	C12—C11—C10	121.50 (14)
C2—C1—H1C	109.5	C12—C11—O3	118.87 (14)
H1A—C1—H1C	109.5	C10—C11—O3	119.49 (12)
H1B—C1—H1C	109.5	C11—C12—C13	119.64 (15)
O1—C2—C3	120.27 (15)	C11—C12—H12	120.2
O1—C2—C1	119.83 (14)	C13—C12—H12	120.2
C3—C2—C1	119.89 (14)	C14—C13—C12	120.25 (14)
C4—C3—C8	118.28 (12)	C14—C13—H13	119.9
C4—C3—C2	118.95 (13)	C12—C13—H13	119.9
C8—C3—C2	122.74 (14)	C13—C14—C15	119.59 (15)
C5—C4—C3	121.56 (13)	C13—C14—H14	120.2
C5—C4—H4	119.2	C15—C14—H14	120.2
C3—C4—H4	119.2	C14—C15—C10	121.16 (15)
C4—C5—C6	119.58 (14)	C14—C15—H15	119.4
C4—C5—H5	120.2	C10—C15—H15	119.4
C6—C5—H5	120.2	O4—C16—O3	123.25 (16)
C7—C6—C5	119.67 (13)	O4—C16—C17	126.61 (17)
C7—C6—N1	118.01 (12)	O3—C16—C17	110.13 (15)
C5—C6—N1	122.28 (13)	C16—C17—H17A	109.5
C6—C7—C8	120.17 (13)	C16—C17—H17B	109.5
C6—C7—H7	119.9	H17A—C17—H17B	109.5
C8—C7—H7	119.9	C16—C17—H17C	109.5
C7—C8—C3	120.73 (14)	H17A—C17—H17C	109.5
C7—C8—H8	119.6	H17B—C17—H17C	109.5
C3—C8—H8	119.6	C9—N1—C6	126.92 (12)
O2—C9—N1	124.00 (13)	C9—N1—H1	116.5
O2—C9—C10	121.78 (13)	C6—N1—H1	116.5
N1—C9—C10	114.22 (12)	C16—O3—C11	116.38 (12)
C11—C10—C15	117.84 (13)		
			
O1—C2—C3—C4	4.4 (2)	C9—C10—C11—C12	176.85 (13)
C1—C2—C3—C4	−174.76 (17)	C15—C10—C11—O3	175.68 (13)
O1—C2—C3—C8	−177.69 (16)	C9—C10—C11—O3	−7.6 (2)
C1—C2—C3—C8	3.1 (2)	C10—C11—C12—C13	−1.0 (2)
C8—C3—C4—C5	−0.4 (2)	O3—C11—C12—C13	−176.59 (14)
C2—C3—C4—C5	177.60 (15)	C11—C12—C13—C14	0.7 (2)
C3—C4—C5—C6	1.1 (3)	C12—C13—C14—C15	0.4 (3)
C4—C5—C6—C7	−1.0 (2)	C13—C14—C15—C10	−1.3 (2)
C4—C5—C6—N1	−178.77 (15)	C11—C10—C15—C14	1.1 (2)
C5—C6—C7—C8	0.2 (3)	C9—C10—C15—C14	−175.65 (14)
N1—C6—C7—C8	178.10 (15)	O2—C9—N1—C6	−1.7 (3)
C6—C7—C8—C3	0.5 (3)	C10—C9—N1—C6	178.01 (13)
C4—C3—C8—C7	−0.4 (3)	C7—C6—N1—C9	156.85 (16)
C2—C3—C8—C7	−178.31 (16)	C5—C6—N1—C9	−25.3 (2)
O2—C9—C10—C11	−43.7 (2)	O4—C16—O3—C11	2.0 (2)
N1—C9—C10—C11	136.62 (15)	C17—C16—O3—C11	−178.92 (13)
O2—C9—C10—C15	132.97 (18)	C12—C11—O3—C16	−85.66 (17)
N1—C9—C10—C15	−46.7 (2)	C10—C11—O3—C16	98.63 (16)
C15—C10—C11—C12	0.1 (2)		

### Hydrogen-bond geometry (Å, º)

*Cg*1 is the centroid of the C10–C15 ring.

**Table:**

*D*—H···*A*	*D*—H	H···*A*	*D*···*A*	*D*—H···*A*
C5—H5···O2	0.93	2.35	2.8792 (19)	116
C12—H12···O4^i^	0.93	2.59	3.396 (2)	145
N1—H1···O1^ii^	0.86	2.08	2.9181 (16)	164
C8—H8···*Cg*1^iii^	0.93	3.20	4.0960 (15)	164

Symmetry codes: (i) −*x*+1, −*y*+2, −*z*; (ii) *x*−1/2, −*y*+1/2, *z*−1/2; (iii) −*x*+1/2, *y*−1/2, −*z*+1/2.
