# Supplementary material for: Synthesis, crystal structure and in vitro anti-proliferative activity of 2-[(4-acetyl­phen­yl)carbamo­yl]phenyl acetate
Source: Acta Crystallogr E Crystallogr Commun. 2023 Oct 5;79(Pt 11):999–1002. doi: 10.1107/S2056989023008526 (PMC10626967; doi:10.1107/S2056989023008526)
Supplement: Supplementary file 4 [file e-79-00999-sup4.docx]

**
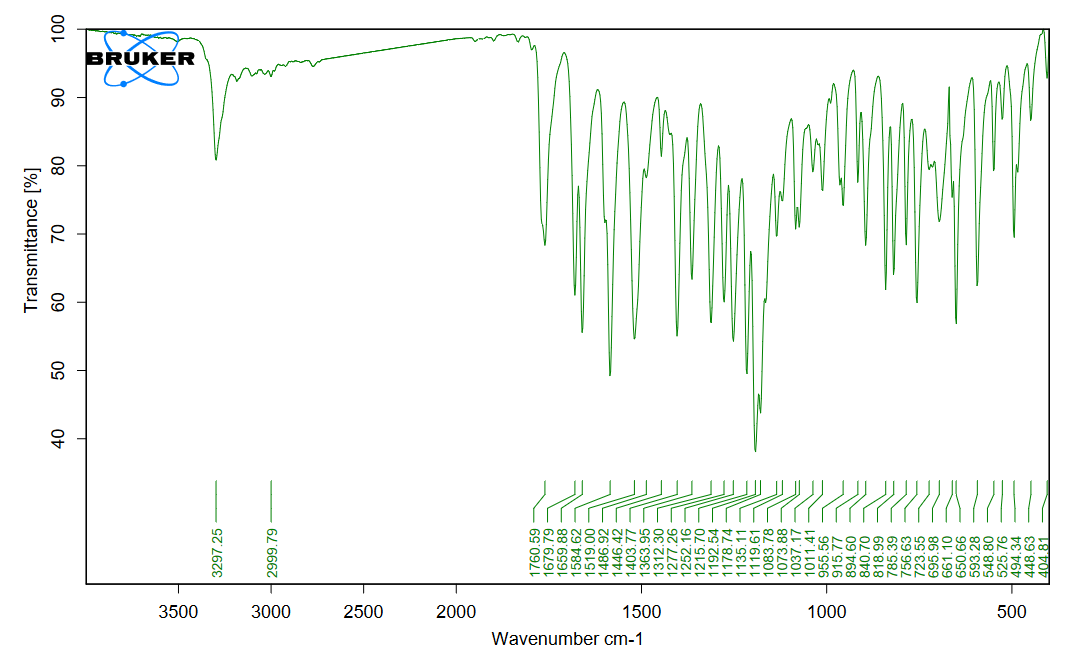
**

**Fig. S1.** IR spectrum of compound **3**


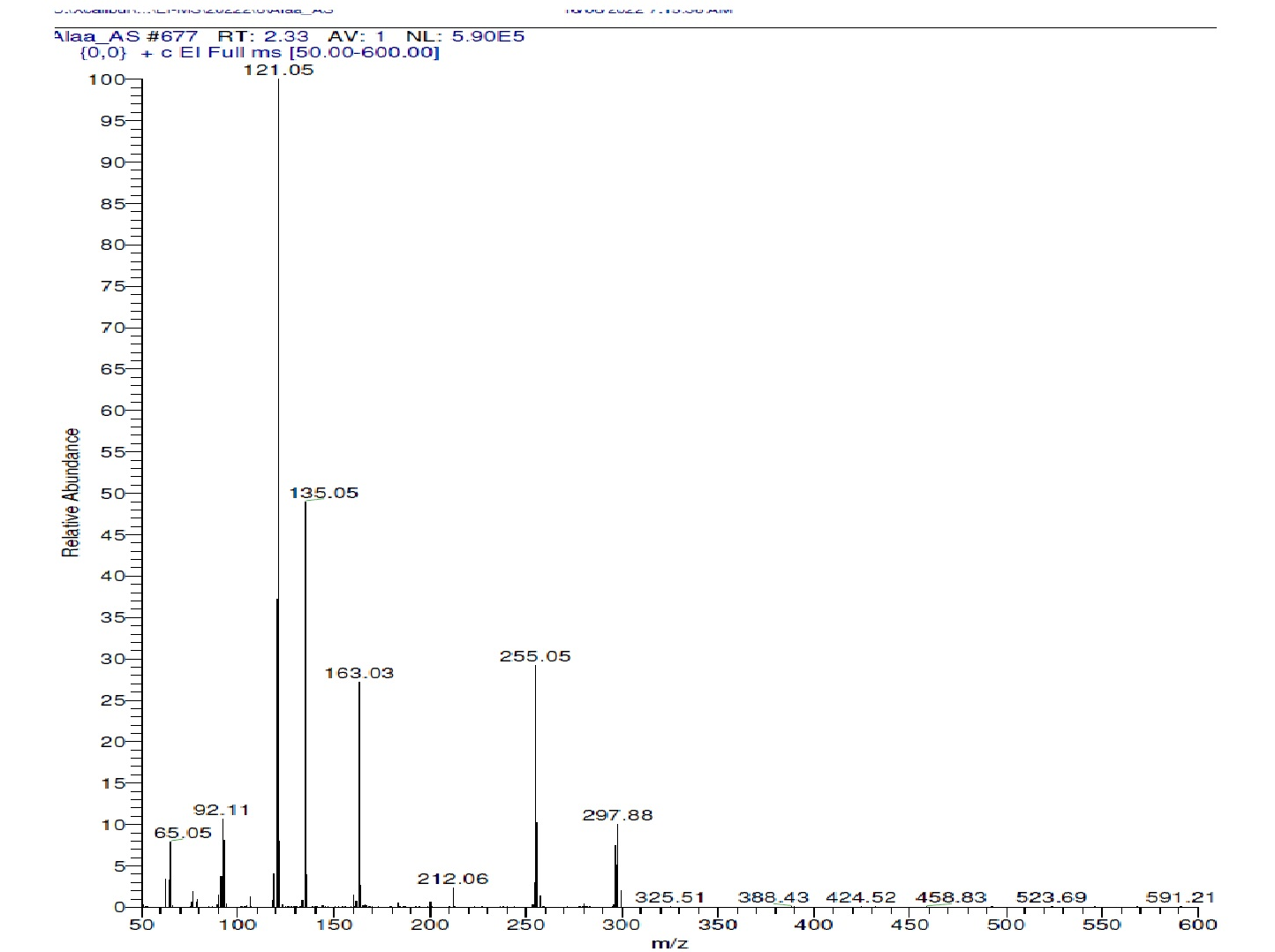


**Fig. S2**. Mass spectrum of compound **3**

**Fig. S3.** ^1^H NMR spectrum of compound **3**

**Fig. S4.** ^13^C NMR spectrum of compound **3**
